# Silane-Treated Basalt Fiber–Reinforced Poly(butylene succinate) Biocomposites: Interfacial Crystallization and Tensile Properties

**DOI:** 10.3390/polym9080351

**Published:** 2017-08-09

**Authors:** Lin Sang, Mingyuan Zhao, Qiushi Liang, Zhiyong Wei

**Affiliations:** 1 School of Automotive Engineering, State Key Laboratory of Structural Analysis for Industrial Equipment, Dalian University of Technology, Dalian 116024, China; sanglin@dlut.edu.cn (L.S.); zhaomingyuan_1111@163.com (M.Z.); liangqiushi@mail.dlut.edu.cn (Q.L.); 2 Department of Polymer Science and Materials, School of Chemical Engineering, Dalian University of Technology, Dalian 116024, China

**Keywords:** composites, crystal structure, mechanical properties, structure-property relations

## Abstract

In this work, an economical modifier silane agent—KH550—was used for surface treatment of basalt fiber. Then, a biodegradable poly(butylene succinate) (PBS)/modified basalt fiber (MBF) biocomposite was successfully developed. The effects of silane treatment and fiber mass content on crystalline structure, isothermal crystallization process and mechanical performance of composites were evaluated. The interfacial crystallization of PBS on the surface of MBF was investigated by using a polarized optical microscope (POM). The transcrystalline (TC) structure could be clearly observed and it grew perpendicular to the surface of MBF, which boosted the nucleation ability on PBS crystallization and the strong interfacial interaction between PBS and silane-treated basalt fiber. Under isothermal crystallization kinetics, the incorporation of basalt fiber enhanced the crystallization rate and reduced the crystallization half-time values of composites compared with that of neat PBS due to a heterogeneous nucleation effect. Furthermore, tensile results confirmed that the presence of MBF could greatly improve the tensile strength and modulus. The predicted interfacial shear strength (IFSS) suggested that an enhancement of interfacial bonding could be realized via interfacial crystallization, which was also verified by SEM images. The PBS/MBF biocomposites can be applied in many fields as a low-cost, lightweight, and biodegradable composite material.

## 1. Introduction

In recent decades, considering environmental issues and the energy crisis, green biodegradable polymeric materials and composites have received more and more attention. Basalt fiber–reinforced plastics (BFRPs) are newly emerging composite materials due to their being recyclable, cost effective, and lightweight [1,2,3,4]. Basalt fiber (BF), produced by abundant natural basalt rocks, is a promising fiber reinforcement, which endows basalt fiber with excellent chemical, weather, and heat resistance [5,6]. Although it possesses similar compositions to glass fiber (GF), basalt fiber exhibits better strength and higher elastic modulus characteristics. Among basalt fiber–reinforced thermoplastics, poly(butylene succinate) (PBS) is an excellent green plastics polymerized from bio-based renewable resources monomers butanediol and succinic acid. Moreover, PBS possesses a variety of desirable properties including biodegradability, renewability, and chemical resistance [7,8,9]. However, few investigations have been performed on the compatibility between PBS matrix and bare BF, which limits the practical application of basalt fiber reinforced PBS composites [10].

Various efforts have been made to improve the fiber/matrix interfacial interaction, including functionalization of matrix, adding compatibilizer, and surface modification of the fibers [11,12,13]. These methods could improve interfacial adhesion, contributing to a certain extent to an efficient stress transferring from the matrix to fibers. Compared with the functionalization of the matrix or using a compatibilizer [11], surface modification of fibers is the most common and simplest method to obtain interfacial interaction. Silane treatment [12,13] is an effective, commercially available, and low cost method for surface modification of fibers without destroy the structure of the fibers themselves or weaken fiber strength.

In fiber-reinforced crystalline or semi-crystalline polymer composites, crystallization behavior has been considerably researched, as it affects both the crystalline structure and crystallization behavior and also influences the mechanical performance of composites. Specially, the development of interfacial crystallization occurring in the interface between fiber and matrix would enhance interfacial adhesion [14,15,16,17,18,19,20]. It is generally accepted that the interfacial crystallization would substantially influence mechanical behavior [21,22,23,24]. Some authors have reported that transcrystalline of matrix polymer (i.e., poly(lactic acid) (PLA) [21], isotatic polypropylene (iPP) [22], or PBS [23]) exhibited a positive effect on the interfacial and mechanical properties. Wang et al. [25] studied transcrystalline effects on sisal fiber-reinforced PLA composites. Zhou et al. [9] directly introduced dopamine as a coupling agent to treat ramie fiber and investigated the interfacial crystallization of PBS. In this literature, interfacial crystallization with different microstructures and morphologies were extensively investigated, and interfacial shear stress (IFSS) was discussed from the point of a single fiber. However, few studies have focused on investigating the effect of interfacial crystallization on mechanical properties in short fiber–reinforced composites at a certain ratio.

In the present paper, we first treated basalt fibers using the economical silane agent KH550 as a modifier and then fabricated PBS/untreated fiber and PBS/modified basalt fiber biocomposites with varying ratios by internal mixer and the injection molding method. The growth of a transcrystalline structure on the surface of silane-treated basalt fiber was observed by polarized optical microscopy (POM), and the isothermal crystallization kinetics of PBS and its composites were analyzed by differential scanning calorimetry (DSC). The effect of silane treatment and fiber loading on the tensile properties of PBS/MBF biocomposites were compared, and tensile fractured topography was observed by scanning electron microscope (SEM). Moreover, a mathematical model was adopted to predict the interfacial shear strength (IFSS), which would provide useful information on the relationship between interfacial strength and mechanical performance.

## 2. Materials and Methods

### 2.1. Materials

Poly(butylene succinate) (PBS, Anqing Hexing Chemical, Ltd., Anqing, China) is a thermoplastic polymer with a melting flow index (MFI) of 15–25 g·10 min^−1^ (150 °C, 2.16 kg) and a density of 1.26 g·cm^−3^. Its melting point is 113 °C, and its number-average molecular weight is 1.7 × 10^5^ g·mol^−1^, as reported by the manufacture. The continuous basalt fibers (GBF 9-800, Shanxi Basalt Fiber Technology Co., Ltd., Taiyuan, China) possess the following characteristics: their density is 2.8 g·cm^−3^, and their tensile strength and tensile modulus is 3.0 and 90–110 GPa, respectively. The silane coupling agent KH550 (NH_2_CH_2_CH_2_CH_2_Si(OC_2_H_5_)_3_), acetone, ethanol (95% purity), and other chemicals used in this study were purchased from Kelong Chemical reagent Ltd., Chengdu, China.

### 2.2. The Silane Treatment of Bbasalt Fiber (BF)

The surface treatment of BF was performed according to previous work [26]. Basalt fibers were first cleaned with acetone/ethanol for 2 h to remove the organic residues from the fiber surfaces. The fibers were repeatedly washed with deionized water to neutrality. After that, BF was immersed in the silane coupling agent (KH550, 0.75 wt %) in water/ethanol for 30 min. Finally, the fibers were reacted and dried in a vacuum oven at 120 °C for 2 h. The silane-treated fibers were marked as MBF.

### 2.3. Preparation of PBS/MBF Biocomposites

The continuous untreated and silane-treated basalt fibers were cut into a settled length of 10 mm. Basalt fibers and PBS granules were dried in a vacuum oven at 80 °C for 12 h to before blending. PBS and silane-treated basalt fibers were compounded via a two-roll mill method. The blending was conducted via the following procedure: First, about half of the PBS was placed inside the mixing chamber at 130 °C for about 1 min at 30 rpm. Then, varying BF (or MBF) content was added over a period of 3 min. Then, the rest of PBS resin was introduced into the mixing chamber. The mixing speed was increased to 60 rpm, and mixing continued for another 8 min. The melting composites were cooled and subsequently chopped to granules.

The resulted granules were dried at 85 °C overnight and then molded using an injection molding machine (XTK 1200, Xiatian General Machinery, Ningbo, China). The temperatures at the three injection regions were 140 °C, 155 °C and 155 °C, respectively, and the nozzle temperature was 145 °C. The fiber contents were set at 2, 5, 10, and 15 wt %, respectively. According to the different loadings of basalt fiber, the injection-molded specimens reinforced with untreated and silane-treated basalt fibers were marked as PBS/MBF (98/2), PBS/BF (95/5), PBS/MBF (95/5), PBS/MBF (90/10), and PBS/MBF (85/15), respectively. PBS/BF (95/5) were prepared and set as control sample.

### 2.4. Characterization

#### 2.4.1. Scanning Electron Microscope (SEM)

The surface morphology of basalt fiber and tensile fractured surface of composites were examined using a field emission scanning electron microscope (S-4800N-FESEM, Zeiss, Oberkochen, Germany). The specimens were pre-coated with a thin gold layer before observation.

#### 2.4.2. X-ray Photoelectron Spectrometer (XPS)

The possible interaction between the fiber and silane agent was examined using an X-ray photoelectron spectrometer (XPS, ESCALABTM 250Xi, Thermo Fisher Scientific, Waltham, MA, USA) focused monochromatized Al Kα radiation (15 kV). The survey spectra were collected over a wide binding energy range from 0 to 1350 eV at a take-off angle of 45°. High resolution spectra of C1s peak were specially recorded.

#### 2.4.3. Polarized Optical Microscope (POM)

The dynamic process of interfacial crystallization morphology during isothermal crystallization on the surface of MBF were in situ observed by a polarized optical microscope (POM, Leica DM4500P, Heidelberg, Germany), which was equipped with a (Linkam THMS600, Linkam Scientific instruments, Tadworth, UK) hot-stage and a digital camera (Leica CCD, Heidelberg, Germany). Composites with varying BF loading were made into sandwiches between two cover slips. Then, they were heated to 130 °C, kept at this temperature for 5 min to allow complete melting, and quickly quenched to 89 °C for isothermal crystallization.

#### 2.4.4. Differential Scanning Calorimetry (DSC)

Isothermal crystallization behavior of PBS, PBS/BF and PBS/MBF composites were performed in a nitrogen atmosphere on a differential scanning calorimeter (DSC1, Mettler-Toledo, Zurich, Switzerland). The samples with 6–8 mg were melted at 130 °C for 5 min to eliminate the previous thermal history and then quick-cooled at a rate of 60 °C·min^−1^ to a series of isothermal crystallization temperatures (*T*_c_). The *T*_c_ was held for a period of time to ensure the completion of crystallization. The heat flows during the crystallization process were recorded for data analysis.

#### 2.4.5. Tensile Properties

Tensile properties were measured according to GB/T1040-92 standard using a universal tester (Sans UTM5105X, Shenzhen, China). The tensile specimens were carried out at room temperature (RT) and about 50% relative humidity with a 10 kN load cell and a cross-head speed of 5 mm·min^−1^. The average values of tensile parameters of PBS and its composites were calculated from five successful samples.

## 3. Results

### 3.1. Modification of Basalt Fiber

**Figure 1:**
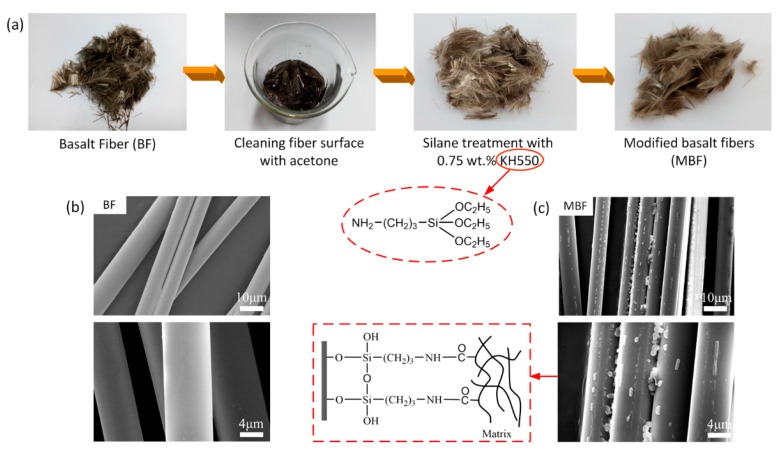
(**a**) The modification process of BF; (**b**,**c**) SEM micrograph of BF and MBF.

The modification process is photographically shown in Figure 1a. KH550 (NH_2_CH_2_CH_2_CH_2_Si(OC_2_H_5_)_3_), an economical organo-silane, was adopted in this study. Silane treatment has proved to be a low-cost and efficient method to endow the fiber-matrix an improved interfacial bonding [26,27,28]. It is clearly visible that raw basalt fibers were brownish in color, whereas the modified basalt fiber (MBF) displayed a light brownish color. During silane treatment, the silane agent first hydrolyzed, formed reactive silanol, and then adsorbed and condensed on the fiber surface. Furthermore, the functional –NH_2_ groups could be connected with the –COOH end groups of PBS by covalent bonds. Thus, after silane treatment, efficient interfacial bonding between the reinforcing element (BF) and the polymeric matrix (PBS) was expected to have been established. To confirm the modification of the basalt fibers, SEM was conducted on the pristine and modified basalt fibers. As shown in Figure 1b,c, a rough surface with small residues was found on the MBF surface, whereas a clean and smooth surface was observed on pristine basalt fiber. This indicated that the silane agent KH550 successfully interacted on the fiber surfaces.

**Figure 2:**
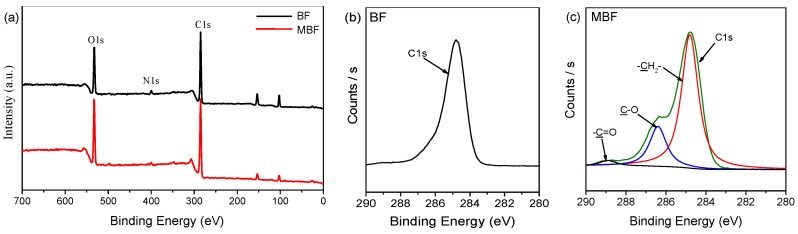
(**a**) X-ray photoelectron spectrometer (XPS) wide scan spectra of basalt fiber (BF) and modified basalt fiber (MBF) with labeled photoelectron (C1s, O1s, and N1s), and XPS high resolutions, C1s spectra of (**b**) BF and (**c**) MBF.

Prior to compounding, XPS was used to detect the elements and interactions after silane treatment of the basalt fibers. As shown in Figure 2a, the XPS wide scan spectra revealed that the presence of carbon and oxygen, and a slight amount of nitrogen could be detected on the surface of BF and MBF. For MBF samples, the relative amount of detected oxygen was increased, which most probably originated from the chemical composition of silane agent. Moreover, the recorded high resolution spectra of the carbon element were evaluated and curve-fitted. The detailed chemical analysis of C1s high resolution scan spectra of BF and MBF are illustrated in Figure 2b,c. The C1s spectra of the MBF can be curve-fitted with three peaks. Beside the aliphatic C–C/C–H peak at 284.8 eV (taken also for charge shift referencing) [29], one is at binding energy (BE) of 286.3 eV for –C–O species and the other is at BE of 288.8 eV for the –C=O species. These rational-derived peaks of C1s attributed to different chemical environment imply that the silane treatment on BF surface was successfully.

### 3.2. MBF-Induced Transcrystallization (TC) of PBS

**Figure 3:**
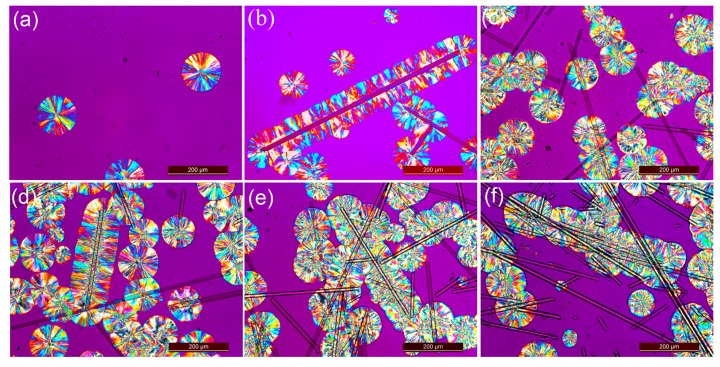
Polarized optical microscope (POM) images of (**a**) PBS, (**b**) PBS/MBF (98/2), (**c**) PBS/BF (95/5), (**d**) PBS/MBF (95/5), (**e**) PBS/MBF (90/10), and (**f**) PBS/MBF (85/15) at 89 °C for 35 min.

In order to investigate the effect of fiber treatment on PBS crystalline morphology, polarized optical microscope (POM) images of crystalline structure at the interface between PBS and MBF are presented in Figure 3. The isothermal crystallization temperature was set at 89 °C for 35 min. For neat PBS, several well-developed spherulites with diameters in a range of 70–100 μm were clearly observed. Figure 3b–f depicted the PBS spherulites of composites in the presence of untreated and silane-treated basalt fiber. For the same crystallization time, more crystals were observed in the composites than the neat PBS, which indicated that the presence of basalt fiber played an accelerating role in the nucleation of PBS. For composites reinforced with pristine BF, no crystallization of PBS on the untreated BF surface was observed (Figure 3c). On the other hand, for PBS/MBF system, a special transcrsytalline (TC) structure was successfully induced on the surface of silane-treated fibers (as shown in Figure 3b). Also, the formation of TC structure was prior to regular PBS spherulites, since some regular spherulites were extremely small. In contrast, the crystallized size of spherulites in PBS/BF (95/5) composites was relatively uniform. This phenomenon indicated that the surface of the silane-treated basalt fibers showed improved heterogeneous nucleation ability for PBS, which can induce the molecular chain to crystallize into orderly structure. However, at a fixed crystallization temperature, a higher density of TC structure was obtained at lower BF loading (i.e., 2 wt % and 5 wt %), while a mixture of spherulites and TC structure was found in higher BF loading (10 wt % and 15 wt %). With increasing amounts of silane-treated fibers, the heterogeneous nucleation effect was expected to be enhanced, which accelerated the formation of spherulites. On the other hand, the number of nucleation sites was also increased, which induced an interfacial crystallization structure. Therefore, one may obtain different crystalline structures in silane-treated fiber reinforced PBS composites by controlling the modified fiber loading.

**Figure 4:**
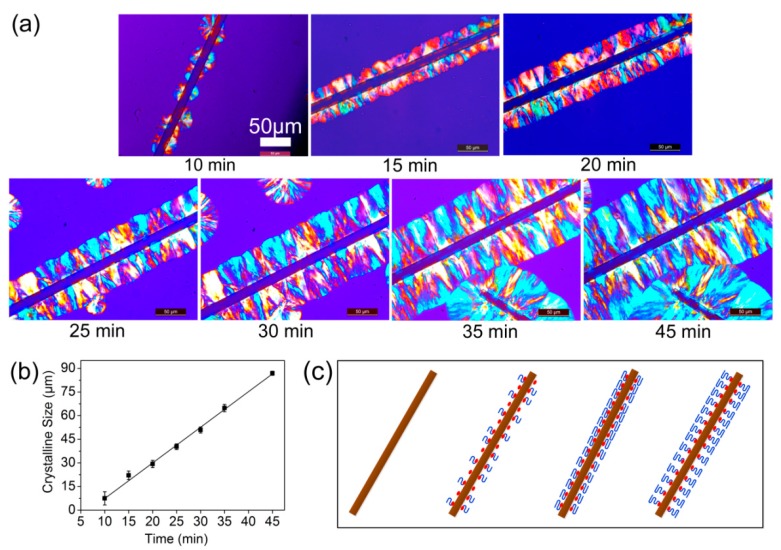
(**a**) The growth of transcrystallization (TC) on MBF of PBS/MBF (98/2) composites crystallized at 89 °C with different time; (**b**) thickness of TC layer; (**c**) schematic illustration of TC structure.

Figure 4 showed the nucleation and growth of the transcrystalline structure of the PBS/MBF (98/2) composite as a representative sample. As shown in Figure 4a, bright interfacial TC structure was gradually formed as the crystallization time increased (10 min → 45 min). After nucleation on the surface for 10 min, interfacial crystallization morphology was observed and identified. However, almost no PBS spherulites were observed far away from the silane-treated fiber. When the crystallization time increased to 15 min, the transcrystalline (lamellae) structure began to grow perpendicular to the silane-treated fiber surface, and the TC structure was formed. As the crystallization time prolonged, the growth of lamella was restricted along the long axis of fiber, and the TC thickness was further increased. By plotting crystallization time and crystalline size, almost a linear relationship can be observed, as shown in Figure 4b. The TC layer on the silane-treated basalt fiber grew to about 8 μm after 10 min and gradually expanded into 87 μm after 45 min. Additionally, the crystalline structure of PBS in the presence of BF is recorded in Supporting Information (Figure S1) during the crystallization process. Comparing the formation processes of crystalline structures, it is predictable that the presence of MBF should be favorable in heterogeneous nucleation and transcrystalline growth compared with that of BF.

Figure 4c illustrates the evolution of TC structure at the interface between the fiber and PBS matrix. The nuclei sites on the surfaces of silane-treated basalt fiber were sufficiently high and distributed randomly, thus the initiated crystallization from the nuclei and the development perpendicular to the fibre direction occurred simultaneously. When the crystallized time increased, the spherulites impinged each other and grew only in one direction due space confinement and finally developed into a transcrystalline region. The formation of TC structure has proven to be beneficial to enhance the interfacial interaction by contributing to effective stress transforming from the matrix to the fiber inside [9,22]. In addition, it has been reported that a rough fiber surface with low surface energy provides abundant heterogeneous nucleation sites to induce transcrystallinity in crystalline polymers [30,31].

### 3.3. Isothermal Crystallization Behaviors

**Figure 5:**
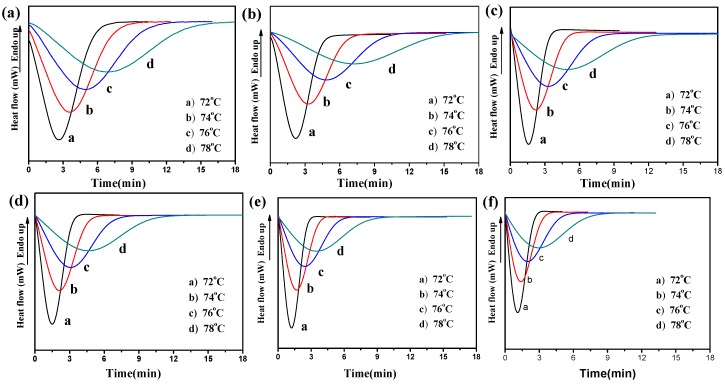
Differential scanning calorimetry (DSC) thermograms of (**a**) PBS, (**b**) PBS/MBF (98/2), (**c**) PBS/BF (95/5), (**d**) PBS/MBF (95/5), (**e**) PBS/MBF (90/10), and (**f**) PBS/MBF (85/15) at different crystallization temperatures.

Differential scanning calorimetry (DSC) was further used to monitor the overall isothermal crystallization process of PBS and its composites at varying crystallization temperatures. By adopting approximate crystallization time, the *T*_c_ interval of PBS and its composites was 72–78 °C. To erase previous thermal history, all composites were quick-cooled to the targeted crystallization temperature (*T*_c_). The DSC crystallization exotherms of neat PBS, PBS/BF, and PBS/MBF composites are shown in Figure 5. As expected, it is observed that as *T*_c_ increased, the exothermal peak became broader for all the samples, suggesting a decreased crystallization rate and an increased time for crystallization completion. On the other hand, the crystallization rate of PBS was obviously enhanced with the increased amount of reinforced fiber.

The well-known Avrami equation was used to track the isothermal crystallization kinetics and PBS and its composites, which contribute to an understanding of the evolution of crystallinity. The equation is listed as follows [32,33]:

(1) $$1-X_{t}=\exp\left(-kt^{n}\right)$$

where *X*_t_ is the relative crystallinity, *t* is the crystallization time, *n* is the Avrami exponent (which is dependent on the mechanism of nucleation and growth geometry), and *k* is the crystallization rate constant. Equation (1) can also be rewritten as a double logarithmic form as follows:

(2) $$\ln[-\ln\left(1-X_{t})\right]=\ln k+n\ln t$$

**Figure 6:**
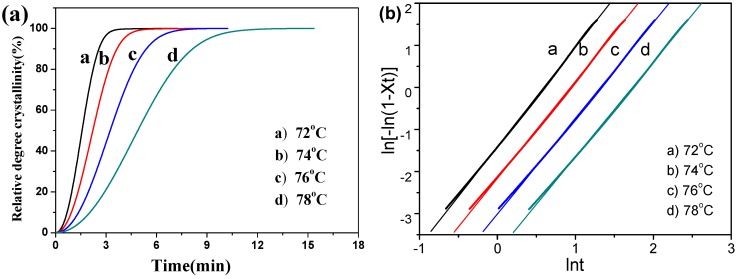
(**a**) Relative degree of crystallinity with time and (**b**) the Avrami plots of ln[−ln(1 − *X*_t_)] versus ln*t* for isothermal crystallization of PBS/MBF (95/5) at different crystallized temperatures.

Figure 6a illustrates the Avrami plots of relative crystallinity (*X*_t_) versus crystallization time (*t*) obtained by exotherm data for the isothermal crystallization at various temperatures (PBS/MBF (95/5) as representative examples). The curves of PBS/MBF (95/5) composites showed S-shaped, which was related to nucleation and the growth process. It was also found that the time of isothermal crystallization completion was strongly dependent on *T*_c_. As illustrated in Figure 6b, a linearity in the Avrami plots was corresponded to crystallinity in a wide range between 5% and 95%. Similar Avrami plots of *X*_t_ and the corresponded linear plots of PBS/BF (95/5) was obtained and shown in Supporting Information (Figure S2). The Avrami parameters *n* and *k* can be obtained from the slope and the intercept of Avrami plots and are summarized in Table 1.

As shown in Table 1, the average *n* values of PBS and its composites ranged from 2.16 to 2.59. For pure PBS with high crystallizability, the *n* values were from 2.40 to 2.58. These values were mainly attributed to a self-nucleation and the homogeneous nucleation mechanisms of PBS. After blending with reinforced fibers, heterogeneous nucleation took place in a predominant way for PBS/MBF composites. For untreated and silane-treated fiber-reinforced composites, the average *n* values of PBS/BF (95/5) and PBS/MBF (95/5) composites were 2.26 and 2.30, respectively. As shown in POM images (Figure 3), there were two different crystalline structures observed in the PBS/MBF composites, including spherulites and transcrystalline layers. Although TC structure was observed in the PBS/MBF composites, transcrystallization was essentially identical with spherulites since these two crystalline structures consist of the same lamellae and the same linear growth rate [25,34,35]. The visual difference between them is that the spherulites began to grow radially from the nuclei, while the transcrystallines grew perpendicular to the surface of silane-treated fibers. Thus, the slight variation of the *n* values indicated that the incorporation of untreated or silane-treated fibers into PBS bulk did not greatly alter the growth dimensionality during the isothermal crystallization process. This might also explain the phenomenon that spherulitic and TC structure co-existed in higher MBF loading composites (observed in Figure 3). At a given crystallization temperature, the *k* values of composites were higher than that of pure PBS, which implies that the heterogeneous nucleation was accelerated by the addition of fibers. As the amount of basalt fiber loading increases, it is also found that the *k* values further increase, which would accelerate the completion of crystallization. As a result, in combination with POM images, it can be concluded that the incorporation of basalt fibers contributed to an improved heterogeneous nucleation ability, and the silane-treated basalt fibers provided abundant nucleation sites to induce transcrystallinity in PBS. However, it is expected that the formation of spherulites or transcrystallinity may not influence the growth rate of PBS crystals.

The half time of crystallization *t*_1/2_, usually defined as the time required to achieve 50% of the final crystallization kinetics, was introduced to evaluate the crystallization rate of crystalline or semi-crystalline polymers. The *t*_1/2_ values can be obtained as the following equation:

(3) $$t_{1/2}={\left(\frac{\ln2}{k}\right)}^{1/n}$$

where *n* and *k* can be achieved according to Equation (3).

The obtained *t*_1/2_ values for PBS and its composites are listed in Table 1. As expected, with increasing crystallization temperature *T*_c_, the *t*_1/2_ value of all the samples correspondingly increased, suggesting a slower crystallization rate. In the presence of untreated and silane-treated basalt fiber, the *t*_1/2_ values became shorter, exhibiting a heterogeneous nucleating effect. Moreover, by increasing the content of silane-treated fiber, the values of *t*_1/2_ further decreased, indicating an accelerated crystallization rate. For example, at a given *T*_c_ of 78 °C, the *t*_1/2_ values of neat PBS is 11.4 min, whereas PBS/BF (95/5) and PBS/MBF (98/2, 95/5, 90/10, and 85/15) completed the crystallization within 5.33, 7.53, 4.88, 3.82, and 3.34 min, respectively.

**Figure 7:**
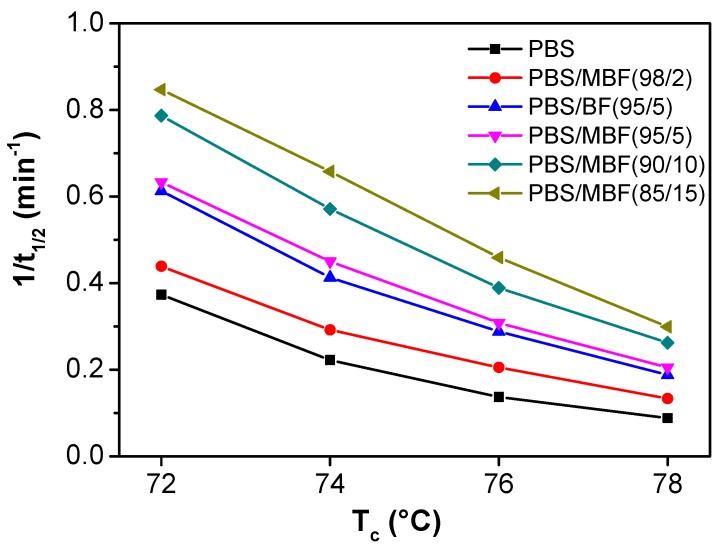
Variation of 1/*t*_1/2_ with *T*_c_ for PBS, PBS/BF and PBS/MBF composites.

Figure 7 illustrates the variation of 1/*t*_1/2_ with *T*_c_ for all of the samples. Conveniently, the reciprocal of *t*_1/2_ represented the crystallization rate. Both PBS and its composites exhibited an increase of 1/*t*_1/2_ values with the reduction of *T*_c_ due to a greater super-cooling at lower *T*_c_, which accelerated the crystallization rate. These enhanced crystallization rates with decreasing *T*_c_ further confirm that the initial nucleation dominated the isothermal crystallization rate of the samples. On the other hand, the 1/*t*_1/2_ value of silane-treated fiber-reinforced PBS composites was greater than that of untreated samples. In particular, the 1/*t*_1/2_ values further prolonged with the increase content of silane-treated basalt fiber. During the crystallization process, the presence of pristine and silane-treated basalt fiber served as nucleating agent for PBS. Furthermore, the silane-treated one presented a better heterogeneous nucleation effect.

### 3.4. Tensile Properties of PBS/MBF Biocomposites

**Figure 8:**
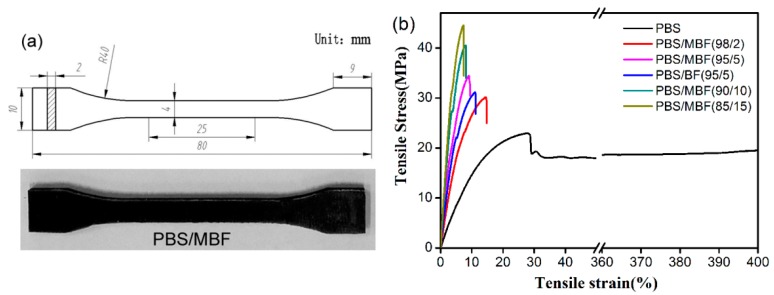
(**a**) Standard tensile samples; (**b**) stress-strain curve of PBS, PBS/BF, and PBS/MBF.

In order to explore the relationship between the transcrystalline structure and macro mechanical performance, the tensile property of composites in accordance with ASTM standards was tested (Figure 8a). The typical stress-strain curves of neat PBS and untreated and silane-treated basalt fiber reinforced composites are shown in Figure 8b and Table 2. PBS exhibited a yielding behavior followed by a cold drawing, which is typical for ductile polymers. On the other hand, the stress-strain behavior of basalt fiber-reinforced composites altered considerably from the neat PBS. The elongation at break decreased dramatically with almost linear tensile behavior. As can be inferred from Table 2, untreated BF and silane-treated BF with same mass loading showed some difference in enhancement in tensile properties. For the PBS matrix, the tensile strength and modulus was 23.2 and 140.6 MPa, respectively. For composites reinforced with pristine basalt fiber (PBS/BF (95/5)), the tensile strength and modulus was enhanced by 7.3 and 126.8 MPa, respectively, which was about 31.5% and 77.7% higher than that of PBS matrix. However, for the PBS/MBF (95/5) biocomposites, tensile strength and modulus were 34.5 and 290.0 MPa, which significantly increased by 48.7% and 144% in contrast to neat PBS, respectively. At a higher silane-treated BF loading, the tensile strength and modulus of PBS/MBF composites were further boosted. For example, as the MBF content increased to 15%, the tensile strength and modulus were increased by 93.1% and 281.3%, compared with neat PBS accordingly. Accordingly, surface modification could contribute to an improved PBS/MBF interfacial compatibility, preventing microvoids and fiber-PBS debonding in the interface region.

**Figure 9:**
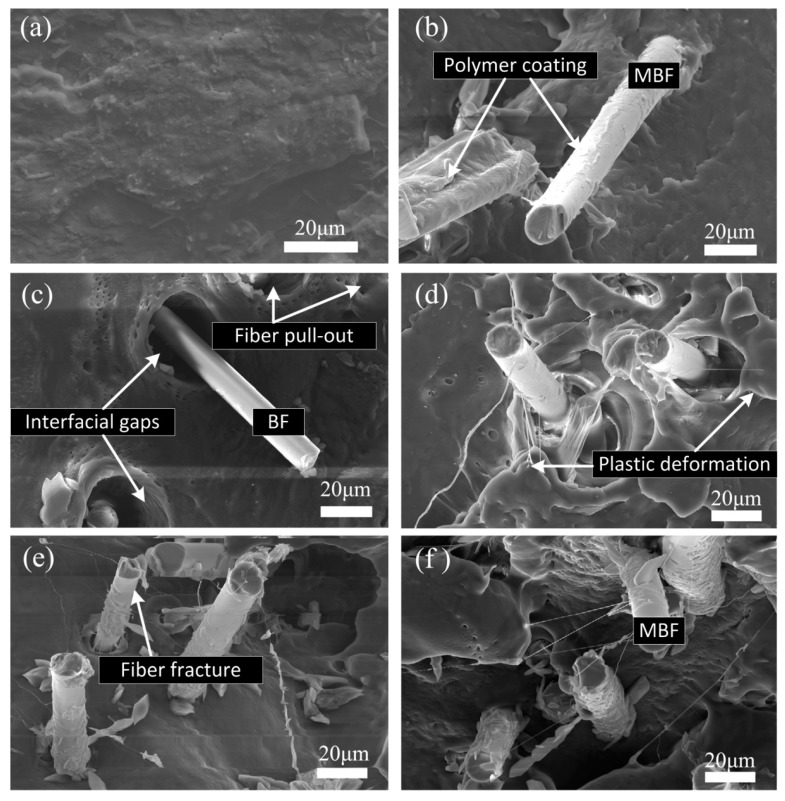
Scanning electron microscopy (SEM) micrographs of tensile fractured surfaces of (**a**) PBS, (**b**) PBS/MBF (98/2), (**c**) PBS/BF (95/5), (**d**) PBS/MBF (95/5), (**e**) PBS/MBF (90/10), and (**f**) PBS/MBF (85/15).

Figure 9 showed the tensile fracture topography of PBS, PBS/BF, and PBS/MBF composites at a high magnification. For PBS/BF (95/5) biocomposites, visible interfacial gaps were found between the fiber and PBS matrix, and fiber-PBS debonding in the interphase region can be clearly observed (Figure 9c), which suggests poor interfacial adhesion between the untreated fibers and PBS. For PBS/MBF composites (Figure 9b,d–f), the polymers are coated on the silane-treated fiber surface, and the voids between PBS matrix and MBF were significantly reduced, suggesting strong interfacial adhesion compared to the untreated fiber.

In order to a obtain deeper understanding on the effect of interfacial crystallization on tensile results and interfacial strength, a simulation of material properties was performed using a mathematical Kelly-Tyson model derived from experimental data of PBS/MBF biocomposites, as given by Equation (4) [36,37].

(4) $$\sigma_{c}=\tau_{i}\left(L/d\right)V_{f}C_{o}+\sigma_{m}V_{m}$$

where *σ*_m_ and *σ*_c_ respectively represent the tensile strength of matrix and its composites, *τ*_i_ represents the interfacial shear strength (ISS), *L* and *d* are the fiber length and diameter, *C*_o_ is the orientation parameter, *V*_m_ and *V*_f_, are the fiber and matrix volume fraction, respectively. Especially, *V*_f_ is calculated by Equation (5) [38].

(5) $$V_{f}=\left[1+\frac{\rho_{f}}{\rho_{m}}\left(\frac{1-W_{f}}{W_{f}}\right)\right]$$

where *ρ*_f_ and *ρ*_m_ are the density of polymer matrix and basalt fiber, respectively. *W*_f_ is the fiber weight fraction in the composite specimen.

Following the Kelly-Tyson model, *τ*_i_ was obtained throughout Equations (4) and (5) and given in Table 2. The average fiber diameter (*d*) and the fiber length (*L*) of composites were obtained from optical microscopy. For short fiber reinforced composites, the empirical value of *C*_o_ for random orientation of fiber in 3D is usually 0.2 [39]. As listed in Table 2, the *τ*_i_ of PBS/BF (95/5) biocomposites was 50.2 MPa, while that of PBS/MBF (95/5) was 76.1 MPa, indicating that the silane treatment would contribute to an improved polymer/fiber interfacial bonding. This is also supported by the observation of interfacial crystallization from POM images as shown in Figure 3 and Figure 4. The higher computed *τ*_i_ of PBS/MBF composites suggested better interfacial bonding between the MBF and PBS matrix. However, the *τ*_i_ of PBS/MBF composites did not increased with the increase content of MBF loading. The highest value of *τ*_i_ of PBS/MBF composites were obtained for PBS/MBF (98/2) biocomposites at a value of 100.1 MPa, which might be due to the more effective formation of interfacial crystallization of PBS to MBF of the composites at lower content of fiber loading. Therefore, the enhanced interfacial adhesion may interpret the improvement of tensile strength of PBS/MBF in contrast with that of PBS/BF composites from the perspective of interfacial crystalline structure.

## 4. Conclusions

Silane treatment was adopted to modify the surface of basalt fibers and improve the interfacial adhesion between PBS matrix and basalt fibers in PBS/MBF biocomposites. It was found that the transcrystalline structure was successfully induced and grew perpendicular to the silane-treated fiber axis, while no TC structure was observed in neat PBS or PBS/BF composites. The overall isothermal crystallization kinetics of PBS with varying silane-treated BF content at different crystallization temperatures was investigated by the Avrami equation. It is observed that the crystallization half-time (1/*t*_1/2_) values of PBS/MBF composites was shorter than that of PBS and its composites reinforced by pristine BF. The silane-treated basalt fiber acted as an efficient nucleating agent and provided abundant nucleating sites, accelerating the completion the crystallization process of PBS. Tensile results showed that the incorporation of silane-treated BF lead to an increase in tensile strength and modulus. The IFSS analysis verified that the interfacial crystalline structure improved PBS/BF interfacial bonding, which was also demonstrated by SEM images. It is concluded that silane treatment of BF may provide an effective method for preparing high-performance and biodegradable composites.

## Figures and Tables

**Table 1:** Avrami parameters of isothermal crystallization kinetics of PBS, PBS/BF, and PBS/MBF.

Sample	*T*_c_ (°C)	*t*_1/2_ (min)	1/*t*_1/2_ (min^−1^)	*k*·(min^−n^)	*n*	Average (*n*)
PBS	72	2.68	0.373	0.054	2.58	2.47
	74	4.50	0.222	0.017	2.47	
	76	7.30	0.137	0.006	2.43	
	78	11.4	0.088	0.002	2.40	
PBS/MBF (98/2)	72	2.28	0.439	0.080	2.67	2.59
	74	3.42	0.292	0.031	2.55	
	76	4.88	0.205	0.013	2.55	
	78	7.53	0.133	0.004	2.58	
PBS/BF (95/5)	72	1.63	0.613	0.232	2.34	2.26
	74	2.32	0.413	0.102	2.33	
	76	3.47	0.288	0.046	2.20	
	78	5.33	0.188	0.020	2.17	
PBS/MBF (95/5)	72	1.58	0.633	0.245	2.36	2.30
	74	2.22	0.45	0.115	2.30	
	76	3.25	0.308	0.049	2.27	
	78	4.88	0.205	0.020	2.25	
PBS/MBF (90/10)	72	1.27	0.787	0.420	2.36	2.26
	74	1.75	0.571	0.201	2.28	
	76	2.57	0.389	0.088	2.23	
	78	3.82	0.262	0.039	2.16	
PBS/MBF (85/15)	72	1.18	0.847	0.499	2.26	2.16
	74	1.52	0.658	0.293	2.16	
	76	2.18	0.459	0.137	2.12	
	78	3.34	0.299	0.058	2.09	

**Table 2:** IFSS of PBS/BF and PBS/MBF composites.

Sample	*σ*_m_ (MPa)	*σ*_ct_ (MPa)	*E* (MPa)	*W*_f_ (%)	*V*_f_ (%)	*L* (μm)	*τ*_i_ (MPa)
PBS/MBF (98/2)	23.2	29.8 ± 1.1	163.2 ± 5.6	2	1	317.3	100.1
PBS/BF (95/5)	23.2	30.5 ± 0.5	267.4 ± 32.5	5	2.3	315.5	50.2
PBS/MBF (95/5)	23.2	34.5 ± 0.3	290.0 ± 19.6	5	2.3	314.2	76.1
PBS/MBF (90/10)	23.2	39.6 ± 0.7	373.9 ± 22.3	10	4.8	307.3	55.2
PBS/MBF (85/15)	23.2	45.8 ± 1.0	442.3 ± 16.8	15	7.3	303.2	51.0

## Supplementary Materials

The following are available online at www.mdpi.com/2073-4360/9/8/351/s1, Figure S1: The growth of polymeric matrix (PBS) spherulites in the presence of pristine basalt fibers crystallized at 89 °C at different times; Figure S2: (a) Relative degree of crystallinity with time and (b) the Avrami plots of ln[−ln(1 − *X*_t_)] versus ln*t* for isothermal crystallization of PBS/BF (95/5) at different crystallized temperatures.
