# Supplementary material for: Silane-Treated Basalt Fiber–Reinforced Poly(butylene succinate) Biocomposites: Interfacial Crystallization and Tensile Properties
Source: Polymers (Basel). 2017 Aug 9;9(8):351. doi: 10.3390/polym9080351 (PMC6418722; doi:10.3390/polym9080351)
Supplement: Supplementary file 1 [file polymers-09-00351-s001.pdf]

Supporting Information

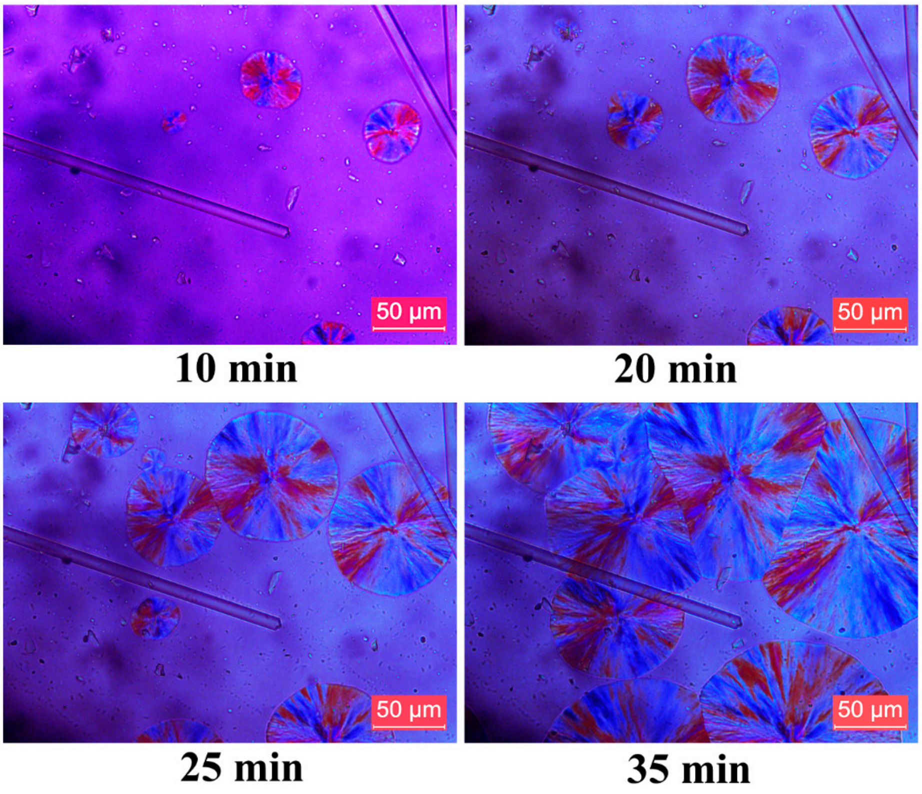

Figure S1. The growth of polymeric matrix (PBS) spherulites in the presence of pristine basalt fibers crystallized at 89 °C at different times.

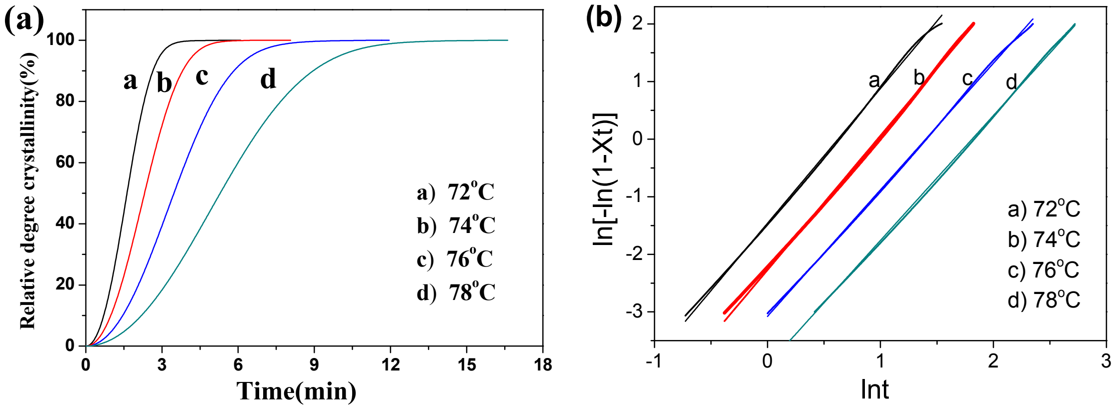

Figure S2. (**a**) Relative degree of crystallinity with time and (**b**) the Avrami plots of ln[-ln(1-X_t)] versus *lnt* for isothermal crystallization of PBS/BF(95/5) at different crystallized temperatures.
